# Early Life Programming of Skeletal Health

**DOI:** 10.1007/s11914-023-00800-y

**Published:** 2023-06-19

**Authors:** Rebecca J. Moon, Natasha L. Citeroni, Riagbonse R. Aihie, Nicholas C. Harvey

**Affiliations:** 1 MRC Lifecourse Epidemiology Centre, University of Southampton, Tremona Road, Southampton, SO16 6YD UK; 2 Paediatric Endocrinology, University Hospital Southampton NHS Foundation Trust, Southampton, UK; 3 Faculty of Medicine, University of Southampton, Southampton, UK; 4 NIHR Southampton Biomedical Research Centre, University of Southampton and University Hospital Southampton NHS Foundation Trust, Southampton, UK

**Keywords:** Osteoporosis, Epidemiology, Bone mineral density, Vitamin D, Calcium, Developmental programming, Epigenetics

## Abstract

**Purpose of Review:**

Increasing bone mineral accrual during childhood might delay the onset of osteoporosis. We discuss the scientific evidence for early life approaches to optimising skeletal health.

**Recent Findings:**

There is an ever-growing body of evidence from observational studies suggesting associations between early life exposures, particularly during foetal development, and bone mineral density (BMD). The findings of such studies are often heterogeneous, and for some exposures, for example, maternal smoking and alcohol intake in pregnancy or age at conception, intervention studies are not feasible. The most frequently studied exposures in intervention studies are calcium or vitamin D supplementation in pregnancy, which overall suggest positive effects on offspring childhood BMD.

**Summary:**

Maternal calcium and/or vitamin D supplementation during pregnancy appear to have positive effects on offspring BMD during early childhood, but further long-term follow-up is required to demonstrate persistence of the effect into later life.

## Introduction

Osteoporosis is a disorder of bone mass and microarchitecture resulting in increased propensity to fracture. Osteoporosis and the associated fragility fractures are an important cause of healthcare use and expenditure. It was estimated that in 2019, 32 million individuals in the European Union plus the United Kingdom (UK) and Switzerland (termed EU27 + 2) were living with osteoporosis. There were estimated to be 4.3 million new fragility fractures during that year, equivalent to 11,705 fractures per day [1]. The total cost of new fragility fractures, existing fractures and pharmacological interventions for osteoporosis in the EU27 + 2 was calculated at €56.9 billion, representing approximately 3.5% of healthcare expenditure in these countries [1]. In addition to pain and disability, fracture is associated with an increased risk of death, and nearly 250,000 deaths in the EU27 + 2 in 2019 were directly attributed to fracture [1]. Importantly, with an ageing population observed in most developed countries, the burden of osteoporosis has and will likely continue to increase unless primary preventative strategies can be implemented. One approach might be to improve skeletal health in early life to improve peak bone mass (PBM) and delay the onset of osteoporosis [2].

## Bone Mass Throughout the Lifecourse

Osteoporotic bone typically has both low bone mineral density (BMD) and a deterioration in the microarchitecture of the bone, including cortical thinning and a reduction in trabecular number and thickness. However, the clinical definition of osteoporosis, as defined by the World Health Organisation (WHO), is based solely on a measurement of BMD by dual-energy X-ray absorptiometry (DXA). BMD is compared to data from a reference population of healthy young adult females to generate a standard deviate “*T*-score”, with a *T*-score less than − 2 used to define osteoporosis [3]. Certainly, the clinical relevance of a low BMD is demonstrated by lower BMD in both adults [4] and children [5] who fracture.

Skeletal development begins in utero at 8–12 weeks’ gestation but foetal skeletal calcium accretion peaks during the third trimester of pregnancy. Foetal bone mineral accretion is primarily determined by foetal plasma calcium ion (Ca^2+^) concentration, which is dependent on the active transport of Ca^2+^ from the maternal to foetal circulation by the placenta [6]. Adaptations to maternal calcitropic hormones and increased intestinal calcium absorption during pregnancy help to facilitate this high foetal calcium demand, yet maternal serum Ca^2+^ is maintained within the normal adult range. At birth, bone mineral constitutes approximately 2% of an infant’s body weight [7].

During childhood and adolescence, skeletal growth in both length and width due to constant modelling and remodelling results in bone mineral accrual and an increase in bone mass (the composite of bone mineral content and bone size). Puberty is a critical period for bone mineral accrual, during which whole body bone mass roughly doubles. Notably, the rate of gain in bone mass temporally lags behind peak growth velocity by approximately 6 months, resulting in a period of relative under-mineralisation [8]. This coincides with an increased incidence of fracture during adolescence, observed later in boys than girls and coinciding with the later puberty onset in males [9]. Following final height achievement, bone mineral accrual continues into early adulthood. Recent analysis of cross-sectional data collected during 2005–2014 in the National Health and Nutrition Examination Survey (NHANES) in the United States of America (USA) suggested PBM was attained between 20 and 24 years in males and 19 and 20 years in females depending on skeletal site. PBM is typically attained earlier at the femoral neck and total hip than lumbar spine in both sexes, with no difference by ethnicity or body mass index [10]. Thereafter, bone mass decreases, with an acceleration in bone loss after the menopause in women. Mathematical modelling has demonstrated that achieving a 10% higher PBM will delay the onset of osteoporosis by 13 years [2], highlighting the necessity to optimise early life bone mineral accrual to reduce the burden of osteoporosis and fragility fracture.

## Modifiable Factors Affecting Bone Health in Childhood and Adolescence

There is evidence of moderate to high tracking (maintenance of the relative position of an individual within the population distribution over time) of BMD through childhood, puberty and into young adulthood [11, 12]. This might partly reflect a genetic tendency as osteoporosis has high heritability and numerous loci associated with BMD have been identified in genome-wide association studies (GWAS) [13]. Thus far, single nucleotide polymorphisms have been identified accounting for up to 20% of the variance in adult BMD [13]; whilst further genetic contribution will undoubtedly be identified in future studies, findings thus far raise the possibility of modifiable lifestyle factors influencing BMD in early and later postnatal life.

Physical activity has beneficial effects on the growing skeleton. In childhood and adolescence, higher levels of physical activity are associated with greater BMC, BMD and measures of bone strength by peripheral quantitative computed tomography in both cross-sectional and longitudinal studies [14–17]. Vigorous physical activity has however been associated with higher risk of fracture, probably due to confounding by greater exposure to potential fracture-inducing events [14]. Two randomised controlled trials (RCT) have demonstrated positive effects of a high impact jumping intervention in pre-pubertal children on whole body, lumbar spine and hip BMC with some persistence of the effect several years after cessation of the intervention [18, 19]. Furthermore, observational studies have also demonstrated associations between self-reported physical activity in adolescence and young adulthood and BMD in mid- and older adulthood [20–22]. For example, Zhang et al., using the Hertfordshire Cohort Study, found self-reported physical activity in women at age 18–29 years was associated with higher total hip BMD at age 72–80 years after adjustment for current activity levels [21], highlighting the importance of promoting physical activity in early life and around PBM to reduce osteoporosis risk.

Consideration of nutritional health is also important to maximise bone mineral accrual. This is of particular importance in preterm infants and those born with low birth weight, who often have had the reduced in utero bone mineral accrual as transplacental calcium transfer is maximum in the third trimester. The lower BMD can persist into later childhood and adulthood in those born preterm [23, 24]. Human breast milk is known to have beneficial effects on the risk of neonatal sepsis and complications of prematurity including necrotising enterocolitis and retinopathy of prematurity [25], and there is some evidence to suggest that there are long-term benefits for bone health [26]. These benefits are similarly observed in term infants in observational studies [27]. Fortification of human milk can also increase BMC in preterm infants [28].

Additionally, particular importance should be given to the nutritional status of children with chronic medical conditions that impact on nutritional intake or intestinal absorption, and are as such at increased risk of poor bone health. Despite bone mineral containing over 99% of the body’s total calcium, the benefits of calcium supplementation in children and adolescents on BMC and BMD are unclear. Prevention of dietary calcium and vitamin D deficiency are vital to prevent rickets and symptomatic hypocalcaemia. However, whilst observational studies suggest positive associations between calcium intake and BMC or BMD [17], the findings of RCTs have generally been inconclusive and few have demonstrated persistence of a positive effect after discontinuation of supplementation. A recent meta-analysis of RCTs of calcium supplementation in children and young adults up to 35 years of age suggested a small positive effect on BMD at the femoral neck and whole body with a similar direction of effect at the lumbar spine and total hip but with confidence intervals bounding zero. The effect appeared to be stronger in studies that included only individuals over 20 years of age, although the number of studies was small, and some studies were limited to only women [29]. Similarly, observational studies suggest a non-linear relationship between 25-hydroxyvitamin D [25(OH)D] status and BMD or BMC in childhood [30], and this is supported by a meta-analysis of RCTs of vitamin D supplementation, but RCTs suggest that the effect of vitamin D supplementation on BMD in healthy children is small and may only be of benefit to those with biochemically low 25(OH)D levels [31–33].

## The In Utero Environment and Long-Term Bone Health

There is now increasing recognition that modifiable exposures occurring before birth impact long-term health. In 1986, Barker and colleagues observed a close geographical relationship between infant mortality rates and standardised mortality from cardiovascular disease 65 years later, leading to the “developmental origins of health and disease hypothesis” [34]. Subsequent observations of relationships between birth weight and other non-communicable diseases extended this hypothesis to other clinical outcomes, including skeletal health. Indeed, birthweight is positively associated with bone mass in both young and late adulthood [35]. Whilst birthweight is a proxy for poor intrauterine nutrition, poor intrauterine growth might not result in low birth weight. The importance of in utero growth, as opposed to simply achieved weight at birth, to long-term skeletal health is further supported by data from both the Southampton Women’s Survey (UK) and Generation R (The Netherlands) mother–offspring birth cohort studies, in which measures of foetal growth derived from ultrasound measurements during pregnancy have also shown positive associations with BMD and hip geometry in childhood [36, 37].

## Maternal Lifestyle in Pregnancy and Offspring Bone Health

Maternal health and lifestyle factors are important determinants of intrauterine growth, and their associations with offspring skeletal health have been assessed in observational studies. Smoking, for example, is recognised to impact negatively on foetal growth and birth weight due to detrimental effects on placental function. However, the relationship of maternal smoking with offspring bone health is more complex. In both the Princess Anne Hospital cohort and the SWS, maternal smoking was associated with lower neonatal BMC and BMD [38, 39]. Jones et al. similarly found lower BMD at age 8 years in Australian children born to mothers who smoked in pregnancy [40], but this relationship was no longer evident at age 16 years [41]. Some studies have found maternal smoking in pregnancy to be associated with higher BMD in childhood [37, 42]. A recently published meta-analysis suggested that maternal smoking in pregnancy was associated with lower offspring BMD in childhood/adolescence, but that this relationship was no longer evident after adjustment for current weight [43]. Indeed, it is well recognised that maternal smoking in pregnancy is associated with greater childhood obesity, and obese children tend to have higher BMD. The intricate relationships between poor in utero growth in infants of smokers, postnatal catch-up weight gain and coupled with increased risk of other postnatal risk factors for poor bone development may underlie these differing relationships in observational studies. Interestingly, offspring of women who smoked during pregnancy were shown in a large population-based registry study in Sweden to have higher risk of fracture from age 5 years to young adulthood; however, the risk of fracture in siblings with differing in utero smoking exposure was similar, suggesting this risk might be confounded by similar post-natal environmental characteristics [44], although other shared maternal factors during pregnancy (e.g. diet, body composition) may also be relevant.

Alcohol exposure during foetal life appears to have a detrimental effect on bone development. Children with foetal alcohol spectrum disorders (FASD) have lower WBLH BMD in adolescence than controls [45]. This might reflect their shorter stature given the effect of bone size on BMD measurement by DXA, and greater exposure to other drugs of abuse is seen in children with FASD and might also affect bone health [45]. However, animal studies have suggested that even low levels of alcohol exposure are deleterious to bone development independent of the effect on foetal growth [46]. In a large prospective birth cohort study in Finland, moderate maternal alcohol consumption was associated with a more than two-fold increased risk of fracture before 8 years of age [47], but to our knowledge, there are no studies assessing pregnancy alcohol use and offspring BMD.

In developing countries such as the UK, maternal age at conception is advancing [48], which based on the findings of a birth cohort in Sweden might increase the risk of poor long-term offspring bone health. In that study, increasing maternal age was associated with lower total body and lumbar spine areal BMD in young adult males when controlling for several potential confounders [49]. Maternal parity however does not appear to be associated with offspring BMD [39, 49].

Although physical activity is recognised as important for skeletal development postnatally, the influence of maternal physical activity on offspring bone development has been rarely investigated. In a study of offspring of rats exposed to exercise or control during pregnancy, offspring whole body BMD by DXA did not differ, but offspring tibial cortical volumetric BMD was lower in the offspring of exercised dams [50]. Similarly, in the SWS, walking speed in late pregnancy, used as a measure of maternal physical activity, was negatively associated with neonatal BMC and bone area [39]. These findings raise the possibility of competition between the maternal and foetal skeleton for finite mineral resource.

## Maternal Body Habitus and Offspring Bone Health

The prevalence of maternal obesity is increasing, and is associated with poorer obstetric health [51, 52]. In the SWS, greater maternal triceps skinfold thickness in pregnancy, used as a surrogate marker of adiposity, was associated with greater neonatal BA and BMC [39]. Similarly, in a cohort study in Japan, children born to underweight mothers had lower WBLH BMC and BA, but similar BMD, at age 10 years. However, it is probable that this was mediated by offspring weight, which may also have shared genetic and environmental origins in mother and child [53]. Indeed, there is likely a non-linear relationship between maternal body habitus and offspring bone development, but there is stronger evidence to support a role for dietary quality and components affecting offspring bone health.

## Maternal Nutrition in Pregnancy and Offspring Bone Health

### Dietary Quality

Several studies have examined the relationships between maternal overall dietary quality and offspring bone health. In the Princess Anne Hospital Study, 198 women had diet assessed by a food frequency questionnaire at 15 and 32 weeks’ gestation. A dietary score was generated to quantify dietary intake compared to recommendations for a healthy diet. In late pregnancy, a diet characterised by higher intakes of fruit, vegetables, wholemeal bread, rice and pasta, yoghurt and breakfast cereals and lower intakes of chips, roast potatoes, processed meats, sugar, crisps and soft drinks, termed a “prudent diet”, was positively associated with offspring whole body and lumbar spine BMC and aBMD at 9 years of age, including after adjustment for maternal educational achievement, social class, anthropometry and smoking status [54]. Similarly, in over 50,000 mother–offspring pairs in the Danish National Birth Cohort study, a more Western diet in mid-pregnancy, characterised by high intakes of meat, potatoes and white bread but low fruit and vegetable intake, was associated with higher offspring risk of childhood forearm fracture [55]. More recently published work using the SWS and ALSPAC birth cohorts showed maternal consumption of a diet with a high dietary inflammatory index was negatively associated with offspring BA, BMC and aBMD in childhood [56].

### Individual Dietary Components

Many studies have been undertaken to establish the roles of individual dietary components in bone health. Care must be taken in the interpretation of these due to potential inaccuracies in establishing true intakes from food frequency questionnaires; blood biomarkers of dietary components may be more reliable. There is also greater potential for intervention studies to establish causality for single micronutrients than overall dietary quality in determining offspring BMD.

#### Calcium

Unsurprisingly, considering the importance of calcium to bone mineral accrual, the role of maternal calcium intake in pregnancy on offspring bone health has received much attention. A recent meta-analysis with literature searches performed in September 2020 identified six RCTs of calcium supplementation in pregnancy with assessment of offspring bone health [57]; a small effect of calcium supplementation on whole body BMD in the neonatal period was noted; however, a long-lasting effect could not be established from the available studies.

#### Polyunsaturated Fatty Acids (PUFA)

Data from animal models suggest that PUFA, derived from fish oils, have a role in bone metabolism [58, 59]. In the SWS, maternal plasma n-3 PUFA at 34 weeks’ gestation was positively associated with offspring WBLH and LS BMC and BMD at 4 years of age [60]. In one recently published RCT, fish-oil supplementation during pregnancy increased offspring WBLH BMC but not BMD at age 6 years. BMI, fat mass and lean mass were also higher in the fish-oil supplemented offspring suggesting a general growth stimulating effect rather than specific to bone [61].

#### Iron

Fibroblast growth factor-23 (FGF23) regulates body phosphate homeostasis, primarily by increasing phosphaturia, and is also involved in vitamin D regulation. Maternal iron deficiency is associated with increased expression of FGF23 and has therefore been postulated to have implications for the developing skeleton. Antenatal iron supplementation has recently been shown to reduce maternal and neonatal FGF23 [62], but a direct effect on bone health has not yet been examined.

#### Vitamin A

Vitamin A comprises a group of fat-soluble essential nutrients that have roles in vision, immune function, growth and cell division and differentiation. In older adults, the different forms of dietary vitamin A have opposing associations with fracture risk [63]: high intakes of retinol, primarily from animal sources, are associated with high fracture risk, whereas β-carotene intake, primarily from plant sources, is negatively associated with fracture risk. Similarly, in the SWS birth cohort, maternal serum retinol in late pregnancy was negatively associated with offspring neonatal whole body BMC and BA, but not BMD, whereas maternal serum β-carotene concentration was positively associated with BMC and BA but again not BMD [64]. Comparably, in a much smaller mother–offspring study in Norway, maternal serum retinol in pregnancy was not associated with offspring whole body or LS BMD at age 26 years [65]. Overall, these findings lend support to dietary recommendations to limit retinol intake during pregnancy due to the known teratogenic effects, and suggest possible beneficial effects of carotenoids on offspring bone health should be investigated.

#### Folate

Folate supplementation during pregnancy is strongly advised to prevent neural tube defects. Observational studies assessing the relationships between maternal folate intake and offspring BMD are conflicting [66–68], but dose-comparator intervention studies on this outcome are lacking.

## Maternal Vitamin D Status in Pregnancy and Offspring Bone Health

Vitamin D is primarily obtained from the action of sunlight on the skin rather than through dietary sources. However, vitamin D deficiency is highly prevalent in pregnant women [69] and can be improved with supplementation and/or food fortification practices [70]. Considering this and the importance of 25(OH)D in maternal calcium absorption, the effect of maternal vitamin D supplementation on offspring BMD has received scientific interest. Observational studies assessing the associations between maternal 25(OH)D status and offspring BMD have reported inconsistent findings, but variation in gestation at 25(OH)D assessment, age at follow-up, cohort demographics and geographical location of the study limit comparison between studies (Table 1) [71–89].

**Table 1:** Observational studies of maternal and/or umbilical cord blood vitamin D status and offspring assessment of bone health

Study	Country (cohort name)	Number of mother–offspring pairs	Gestation at 25(OH)D assessment	Age at offspring assessment	Method (site) of bone assessment	Association between maternal or cord 25(OH)D levels and bone outcomes
Velkarvh, 2019 [83]	Slovenia (My-MILK project)	73	Third trimester	0–2 days	QUS (tibia)	No association with tibial speed of sound
Levkovitz 2022 [84]	Israel	74	In labour and umbilical cord blood	0–2 days	QUS (tibia)	No association with speed of sound
Namgung, 1998 [74]	South Korea	71	Umbilical cord blood	0–3 days	DXA (whole body)	Positive association with whole body BMC (*r* = 0.24, *p* = 0.047) 6% greater whole body BMC in summer-born compared to winter-born infants
Liao, 2010 [85]	China	32	During labour and umbilical cord blood	0–6 days	QUS (tibia)	Positive associations between both maternal (*r* = 0.40, *p* = 0.02) and umbilical cord (*r* = 0.39, *p* = 0.03) 25(OH)D and offspring tibia speed of sound Higher speed of sound in summer- compared to winter-born infants
Boghossian, 2019 [86]	USA (Calcium for Preeclampsia Prevention trial)	252	11–21 weeks, 26–29 weeks and 36 weeks	0–7 days	DXA (whole body and lumbar spine)	Maternal vitamin D deficiency associated with lower whole body BMD
Weiler, 2005 [87]	Canada	50	Within 48 h of delivery	0–15 days	DXA (whole body, lumbar spine and whole femur)	Whole body BMC/body weight and whole femur BMC/body weight both greater in infants born to vitamin D sufficient compared with deficient mothers
Vilajakainen, 2010 [82]	Finland	98	Mean of two measurements collected during the first trimester and at 2 days postpartum	Average 10 days	pQCT (tibia)	BMC and bone cross-sectional area but not BMD higher in offspring of mothers with 25(OH)D above study cohort median
Dror, 2012 [88]	USA	80	In labour and umbilical cord blood	8–21 days	DXA (whole body)	25(OH)D not associated with whole body BMC in multivariate models
Prentice, 2009 [89]	Gambia	125	20 and 36 weeks	2 weeks, 13 weeks and 52 weeks	SPA (mid-shaft radius), DXA (whole body) in a subset	No associations identified between 25(OH)D at either gestation and measures of BMC/BMD/bone area by either SPA or DXA at any age
Viljakainen, 2011 [81]	Finland	68	Mean of two measurements collected during the first trimester and at 2 days postpartum	14 months	pQCT (tibia)	Bone CSA greater in children born to mothers with higher than median for the study group 25(OH)D but no difference in BMC or BMD
Garcia, 2017 [75]	Netherlands (Generation R study)	4815	During mid-pregnancy (median (IQR) 20·4 weeks (19·9–21·1))	6 years	DXA (WBLH)	Negative associations between maternal 25(OH)D and offspring WBLH BMC, BMD and bone area
Moon, 2015 [72]	UK (Southampton Women’s Survey)	1030	34 weeks	6–7 years	DXA (WBLH and lumbar spine)	Lower WBLH BMC, BMD and bone area and lumbar spine BMC in offspring born to mothers with 25(OH)D < 25 nmol/l compared to the remainder of the cohort
Norgaard 2021 [71]	Denmark (Odense Child Cohort Study)	997	Early pregnancy (< 20 weeks) and late pregnancy (> 20 weeks) and umbilical cord	7 years	DXA (WBLH)	No association with WBLH BMD
Javaid, 2006 [73]	UK (Princess Ann Hospital Cohort)	198	Late pregnancy (mean 34 weeks)	9 years	DXA (whole body and lumbar spine)	Positive association with whole body BMC (*r* = 0.21, *p* = 0.008) and BMD (*r* = 0.21, *p* = 0.008) and lumbar spine BMC (*r* = 0.17, *p* = 0.03) and BMD (*r* = 0.21, *p* = 0.009)
Lawlor, 2013 [76]	UK (Avon Longitudinal Study of Women and Children)	3960	At any point in pregnancy	9–10 years	DXA (whole body and lumbar spine)	No association between 25(OH)D in any of the three pregnancy trimesters and whole body nor lumbar spine BMC
Hyde 2019 [78]	Australia (Vitamin D in Pregnancy Study)	181	< 16 weeks and 28–32 weeks	11 years	DXA (WBLH and lumbar spine)	Positive association between early pregnancy 25(OH)D and WBLH and lumbar spine BMC and BMD in boys, but not girls
Hyde 2017 [79]	Australia (Vitamin D in Pregnancy Study)	181	< 16 weeks and 28–32 weeks	11 years	DXA (lumbar spine	Positive association between early pregnancy 25(OH)D and lumbar spine trabecular bone score (TBS). No association between late pregnancy 25(OH)D and TBS
Hyde, 2021 [80]	Australia (Vitamin D in Pregnancy Study)	168	< 16 weeks and 28–32 weeks	11 years	QUS (calcaneus)	Positive association between early pregnancy 25(OH)D and speed of sound (*r* = 0.17, *p* = 0.03) but not broadband ultrasound attention or stiffness index. No associations with 28–32 weeks’ gestation 25(OH)D
Zhu, 2014 [77]	Australia (Western Australian Pregnancy Cohort (Raine) Study)	341	18 weeks	20 years	DXA (whole body)	Positive association with whole body BMC and BMD

*25(OH)D*, 25-hyrdoxyvitamin D; *BMC*, bone area; *BMD*, bone mineral density; *DXA*, dual-energy X-ray absorptiometry; *pQCT*, peripheral quantitative computed tomography; *QUS*, quantitative ultrasound; *SOS*, speed of sound; *SPA*, single photon absorptiometry; *TBS*, trabecular bone score

**Fig. 1:**
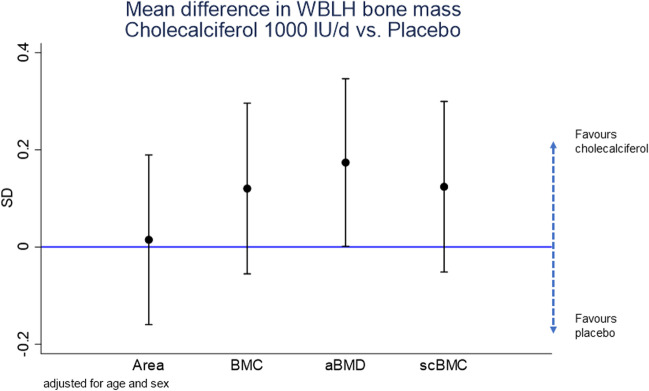
The effect of 1000 IU/day cholecalciferol supplementation from 14 to 17 weeks’ gestation until delivery on offspring whole body less head bone mineralisation at 4 years of age. Reproduced with permission from Curtis et al. JBMR + 2022. BMC, bone mineral content; aBMD, areal bone mineral density; scBMD, size corrected bone mineral content

There have been numerous RCTs that have assessed the effect of pregnancy vitamin D supplementation on neonatal anthropometry and/or calcium status [90]. Our own meta-analysis of these studies suggested a small positive effect on birth weight and neonatal serum calcium. There are, however, fewer studies that have examined the effect of pregnancy vitamin D supplementation on offspring musculoskeletal parameters; to date, there are 2 published trials assessing neonatal BMD [91, 92] and four trials with assessment of BMD in early childhood [93–96]. The largest published RCT is the MAVIDOS study, which was conducted in three centres in the UK and randomised women with a baseline 25(OH)D of 25–100 nmol/l to either 1000 IU/day cholecalciferol or placebo from 14 to 17 weeks’ gestation until delivery [97]. Supplementation increased maternal serum 25(OH)D, although achieved 25(OH)D in late pregnancy was also influenced by maternal BMI, baseline 25(OH)D and genetic variation within the vitamin D metabolism pathway [98–100]. DXA data were obtained for 665 infants within 2 weeks of birth [91] and 452 children at 4 years of age [93]. Across the whole trial population, no effect of vitamin D supplementation on neonatal bone mass was observed, but an interaction with season of birth was noted such that BMD was higher in winter-born infants of supplemented mothers compared to the placebo group [91]. However, at age 4 years, a significant effect of vitamin D supplementation on offspring BMD was noted across the whole cohort [93] (Fig. 1), equating to approximately 0.17 SD difference between the two groups. If this effect is sustained into adulthood, it would be expected to reduce fracture risk. WBLH lean mass was also increased in the cholecalciferol group, consistent with earlier findings from the SWS which showed a positive association between late pregnancy 25(OH)D and offspring grip strength [101].

A positive effect of pregnancy vitamin D supplementation was also found in the Copenhagen Prospective Studies on Asthma in Childhood (COPSAC2010), in which women were randomised to either 2400 IU/day or 400 IU/day cholecalciferol from 24 weeks’ gestation. In that study, differences in WBLH BMC and aBMD at 6 years of age of 0.15 and 0.2 SD, respectively, were identified, thus of comparable magnitude to the differences observed in MAVIDOS [94]. However, no differences in WBLH BMC or aBMD were identified at age 3 years. In contrast, O’Callaghan et al. found no differences in WBLH BMD or BMC at 4 years of age in offspring of children born to mothers randomised to either 4200 IU/week, 16,800 IU/week or 28,000 IU/week cholecalciferol in Bangladesh [95]. The differences in study findings likely reflect methodological differences, including timing of commencing vitamin D supplementation, dosing, daily versus weekly supplementation and the geographical location and population in which the study was performed. This highlights the need to consider generalisability of a study population. Nonetheless, taken together, these studies do suggest a possible benefit of pregnancy vitamin D supplementation for offspring bone health, but further follow-up of these cohorts is needed to demonstrate persistence into adolescence and adulthood.

## Mechanisms

The mechanisms underlying the observed associations between the early environment and future bone health are not fully understood. Animal models allow for more marked dietary restrictions than is typically observed in human studies, and can be used to identify potential mechanisms for the observations. For example, rats fed a protein-deficient diet in pregnant have offspring with fewer ossification centres and alterations to the growth plate [102], and in ewes fed a calorie-restricted diet, mesenchymal stem cell activity was reduced [103].

In humans, there may be a direct effect of individual nutrients or overall nutrition (directly or indirectly due to an effect on placental function, e.g. smoking) on bone mineralisation. Maternal 25(OH)D supplementation for example increases umbilical cord calcium concentration [90], and thus, it could be postulated that this increases calcium availability for skeletal mineralisation. However, interestingly, the winter effect of vitamin D supplementation on neonatal BMD at birth [91], but with a positive effect at age 4 years, regardless of season [93], in MAVIDOS, and similar observations at ages 3 and 6 years in COPSAC2010 [94], suggests an evolving effect of vitamin D supplementation on BMD over childhood. This would support an epigenetic mechanism underlying this relationship. Epigenetics is the study of the interaction between the environment and gene expression. Genes can be differentially expressed in different cells and tissues according to function and need, and in experimental studies, alterations to offspring phenotype and gene expression can occur in response to environmental cues and maternal diet [104]. DNA methylation and histone modification are two types of epigenetic mechanisms. These are stable heritable changes that influence gene transcription without changing the DNA sequence, and can be influenced by the in utero environment, such as maternal smoking [105] and, in the MAVIDOS trial, pregnancy vitamin D supplementation [106]. Differences in the level of DNA methylation have been associated with childhood bone mass [107, 108], with methylation at the *retinoid-X-receptor-a (RXRA)* gene in perinatal tissue implicated as a potential link between maternal 25-hydroxyvitamin D status, vitamin D supplementation and offspring bone mass in both observational and intervention settings [106, 107].

## Conclusions

Optimisation of bone mineral accrual during early life is likely to reduce the risk and delay the onset of osteoporosis in later life. Approaches to this include during childhood, such as physical activity and nutrition, and during in utero development. Recently, pregnancy vitamin D supplementation has been demonstrated to have promising beneficial effects on offspring BMD in early childhood in two large randomised controlled trials in the UK [91, 93] and Denmark [94], and a meta-analysis has suggested a small effect of calcium supplementation in pregnancy on neonatal BMD [57]. Ongoing follow-up of these children is required to show persistence of this effect into later life, and further epigenetic and metabolic studies might help to elucidate the underlying mechanisms of this effect.
